# Supplementary material for: Detailed molecular and epigenetic characterization of the pig IPEC-J2 and chicken SL-29 cell lines
Source: iScience. 2023 Feb 20;26(3):106252. doi: 10.1016/j.isci.2023.106252 (PMC10018572; doi:10.1016/j.isci.2023.106252)
Supplement: Data S2. Complete homer output for identified motifs in Chicken SL-29, related to Tables 5 and 6 — Homer motif analysis results for histone modifications H3K4me1, H3K4me3, H3K27ac, enhancers, and ATAC-seq of chicken SL-29 cell line. Parameters for possible false positives is as mentioned earlier for S5. [file mmc3.zip › Data_S2/S6/Chicken_SL_29/peak_files_ATAC/homerResults.html]

peak\_files/ - Homer de novo Motif Results


# Homer *de novo* Motif Results (peak\_files/)

Known Motif Enrichment Results  
Gene Ontology Enrichment Results  
If Homer is having trouble matching a motif to a known motif, try copy/pasting the matrix file into
STAMP  
More information on motif finding results: HOMER
| Description of Results
| Tips
  
Total target sequences = 86954  
Total background sequences = 86200  
\* - possible false positive  

|  |  |  |  |  |  |  |  |  |
| --- | --- | --- | --- | --- | --- | --- | --- | --- |
| Rank | Motif | P-value | log P-pvalue | % of Targets | % of Background | STD(Bg STD) | Best Match/Details | Motif File |
| 1 | C G T A G A T C A C T G A T G C C T A G C A G T T C A G C G T A G A C T G C A T | 1e-1706 | -3.930e+03 | 1.74% | 0.05% | 84.5bp (69.9bp) | PAX6/MA0069.1/Jaspar(0.662) More Information \| Similar Motifs Found | motif file (matrix) |
| 2 | A C T G C G T A A C G T A C G T A G C T A C G T A G T C A C T G | 1e-1220 | -2.810e+03 | 1.78% | 0.12% | 82.8bp (69.8bp) | IRF6/MA1509.1/Jaspar(0.663) More Information \| Similar Motifs Found | motif file (matrix) |
| 3 | A C G T C G T A C G T A C G T A G C A T A C T G A C T G A C T G A C T G A C T G | 1e-1184 | -2.728e+03 | 1.62% | 0.09% | 83.7bp (64.0bp) | PB0201.1\_Zfp281\_2/Jaspar(0.722) More Information \| Similar Motifs Found | motif file (matrix) |
| 4 | A C G T A C G T A C G T A G T C A C G T A G T C A C G T A C G T A G T C A C T G | 1e-1081 | -2.490e+03 | 1.28% | 0.05% | 83.2bp (61.4bp) | PRDM1/MA0508.3/Jaspar(0.759) More Information \| Similar Motifs Found | motif file (matrix) |
| 5 | C G T A C G T A C G T A C G T A A G T C A C T G A G T C A C T G | 1e-697 | -1.606e+03 | 2.06% | 0.37% | 83.2bp (78.2bp) | PRDM15(Zf)/ESC-Prdm15-ChIP-Seq(GSE73694)/Homer(0.626) More Information \| Similar Motifs Found | motif file (matrix) |
| 6 | G A C T A T G C T A C G C G T A T A C G A C G T G T C A A G T C G C A T C A T G | 1e-464 | -1.069e+03 | 6.81% | 3.56% | 87.3bp (95.9bp) | PB0152.1\_Nkx3-1\_2/Jaspar(0.723) More Information \| Similar Motifs Found | motif file (matrix) |
| 7 | A G C T C A T G T C A G T A C G T A G C A G C T G C A T T A C G G C A T A C G T | 1e-393 | -9.056e+02 | 7.81% | 4.52% | 82.6bp (93.6bp) | SF1(NR)/H295R-Nr5a1-ChIP-Seq(GSE44220)/Homer(0.677) More Information \| Similar Motifs Found | motif file (matrix) |
| 8 | T C A G C A T G C T G A C T G A T C A G T C A G A G T C T A G C G A C T G T A C | 1e-376 | -8.676e+02 | 11.27% | 7.33% | 86.2bp (93.2bp) | Zac1(Zf)/Neuro2A-Plagl1-ChIP-Seq(GSE75942)/Homer(0.697) More Information \| Similar Motifs Found | motif file (matrix) |
| 9 | A C T G A G C T A G T C A G T C C G T A A C T G C G T A A C T G | 1e-335 | -7.721e+02 | 12.44% | 8.51% | 79.1bp (90.2bp) | ZBTB26/MA1579.1/Jaspar(0.754) More Information \| Similar Motifs Found | motif file (matrix) |
| 10 | A G T C A C G T T A G C A C G T C G T A A G T C C G A T A G T C C G T A C T A G | 1e-329 | -7.599e+02 | 4.53% | 2.30% | 87.7bp (94.6bp) | ZSCAN29/MA1602.1/Jaspar(0.664) More Information \| Similar Motifs Found | motif file (matrix) |
| 11 | C G T A A C T G A C T G A C T G G C T A C T A G A T C G A C G T | 1e-290 | -6.694e+02 | 16.46% | 12.23% | 85.9bp (90.8bp) | ZNF263/MA0528.2/Jaspar(0.685) More Information \| Similar Motifs Found | motif file (matrix) |
| 12 | A C G T A C T G A C G T A G C T G A T C C G T A C T A G A G C T | 1e-263 | -6.076e+02 | 13.41% | 9.74% | 82.0bp (92.3bp) | ZBTB32/MA1580.1/Jaspar(0.759) More Information \| Similar Motifs Found | motif file (matrix) |
| 13 | A C G T C T G A A C T G A G T C A T G C C G T A A C T G A G T C C T G A A C T G | 1e-253 | -5.847e+02 | 6.19% | 3.79% | 84.0bp (92.3bp) | Zic1::Zic2/MA1628.1/Jaspar(0.669) More Information \| Similar Motifs Found | motif file (matrix) |
| 14 | A C G T A C G T C T G A G C A T T C G A A T C G G C A T C A T G C T G A C A T G | 1e-251 | -5.783e+02 | 4.04% | 2.17% | 85.8bp (97.7bp) | POU6F1(var.2)/MA1549.1/Jaspar(0.640) More Information \| Similar Motifs Found | motif file (matrix) |
| 15 | C G T A A C T G C G A T C A G T A G C T G A C T A T C G T A C G A C T G G T A C | 1e-217 | -5.006e+02 | 2.66% | 1.28% | 81.4bp (93.6bp) | IRF6/MA1509.1/Jaspar(0.646) More Information \| Similar Motifs Found | motif file (matrix) |
| 16 | A C G T C G T A C G A T G T A C G T C A A G T C A C G T C G T A | 1e-190 | -4.392e+02 | 3.01% | 1.61% | 88.3bp (100.2bp) | HMBOX1/MA0895.1/Jaspar(0.654) More Information \| Similar Motifs Found | motif file (matrix) |
| 17 | C G A T G A T C A G T C A G T C A G T C A C G T A T G C C G T A | 1e-158 | -3.650e+02 | 12.55% | 9.74% | 85.3bp (90.9bp) | THAP1/MA0597.1/Jaspar(0.694) More Information \| Similar Motifs Found | motif file (matrix) |
| 18 | A G T C A C G T A C G T A G T C A C T G C G T A C G T A C G T A | 1e-147 | -3.387e+02 | 0.53% | 0.12% | 94.6bp (75.4bp) | POL008.1\_DCE\_S\_I/Jaspar(0.686) More Information \| Similar Motifs Found | motif file (matrix) |
| 19 | A C T G T G C A A C G T A T G C T G C A C G A T A G T C G C T A | 1e-77 | -1.781e+02 | 4.29% | 3.13% | 80.1bp (91.9bp) | PH0017.1\_Cux1\_2/Jaspar(0.817) More Information \| Similar Motifs Found | motif file (matrix) |
| 20 \* | C G T A A C G T A C G T A C G T C G T A A G T C C G T A C G T A A C G T A C T G | 1e-9 | -2.223e+01 | 0.06% | 0.02% | 83.1bp (84.3bp) | SOX2/MA0143.4/Jaspar(0.755) More Information \| Similar Motifs Found | motif file (matrix) |
| 21 \* | A C G T C G T A A C G T A C T G A C T G C G T A A C G T A C G T | 1e-5 | -1.327e+01 | 0.60% | 0.48% | 83.4bp (94.1bp) | ONECUT2/MA0756.1/Jaspar(0.795) More Information \| Similar Motifs Found | motif file (matrix) |
| 22 \* | A G T C A G T C A C G T A C G T A C G T A G T C A G T C A C G T A C G T A C G T | 1e-1 | -3.393e+00 | 0.29% | 0.26% | 76.3bp (95.0bp) | T1ISRE(IRF)/ThioMac-Ifnb-Expression/Homer(0.704) More Information \| Similar Motifs Found | motif file (matrix) |
